# Realistic and inexpensive ultrasound phantoms to demonstrate aortic aneurysm and aortic dissection

**DOI:** 10.1002/ajum.12309

**Published:** 2022-07-25

**Authors:** Kimberly M. Rathbun, Claire F. Harryman, Corey Moore

**Affiliations:** ^1^ Department of Emergency Medicine Augusta University/University of Georgia Medical Partnership Athens Georgia USA; ^2^ Department of Emergency Medicine The Brody School of Medicine at East Carolina University Greenville North Carolina USA

**Keywords:** aortic aneurysm, aortic dissection, education, phantom, simulation

## Abstract

**Introduction:**

Using ultrasound to evaluate for the presence of aortic pathology is a common procedure in the emergency department. Phantoms are models that are used to simulate clinical conditions for teaching ultrasound‐related skills. To date, no ‘homemade’ phantom has been created to model aortic aneurysms, and no phantoms exist to model aortic dissection.

**Methods:**

We used several readily available, inexpensive ingredients to create ultrasound phantoms.

**Results:**

These phantoms realistically mimic aortic aneurysm and aortic dissection.

**Discussion:**

These are the first ‘homemade’ phantoms that demonstrate aortic pathology.

**Conclusions:**

We have created realistic, affordable, easily reproducible phantoms for use in teaching clinicians to use ultrasound when evaluating patients for aortic aneurysm and/or aortic dissection.

## Introduction

**Figure 1:**
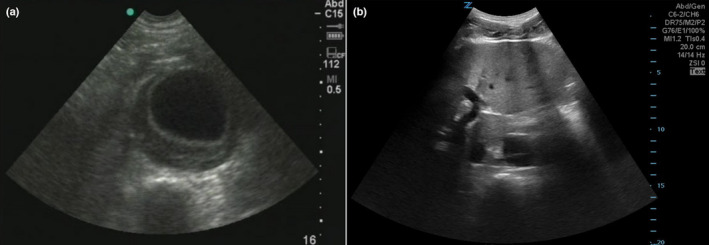
Ultrasound images of aortic aneurysm (a) and aortic dissection (b) in transverse using a low‐frequency curvilinear probe. [Colour figure can be viewed at wileyonlinelibrary.com]

Aortic pathologies, such as a leaking or ruptured aortic aneurysm or aortic dissection, are life‐threatening emergencies that require a timely diagnosis. An aortic aneurysm is a dilation of the aorta that results in the weakening of a part of the aortic wall. A section of the aorta with a diameter of more than 3 cm is defined as an aortic aneurysm (Figure 1a).^1^ Aneurysms can occur anywhere along the aorta but are more common in the abdominal aorta than in the thoracic aorta.^2^ An abdominal aortic aneurysm (AAA) can be found in 4–8% of the US population, but many of these are relatively small (<5.5 cm) and not likely to rupture.^3^, ^4^, ^5^, ^6^, ^7^ Rupture of AAA results in 85–90% mortality.^8^, ^9^ An aortic dissection results from a tear in the aortic intima that then allows blood to flow into the aortic media, resulting in the separation of the vessel wall layers and the creation of a false lumen (Figure 1b). Aortic dissection can occur anywhere along the aorta with the ascending aorta being the most common location and an isolated abdominal aortic dissection being quite rare.^10^ The incidence of aortic dissection ranges from 2.6 to 3.5 per 100,000 person‐years in the general population.^11^, ^12^, ^13^, ^14^ Acute aortic dissection is often fatal with a mortality rate >50–75%.^15^, ^16^

Traditionally, the diagnosis of AAA and aortic dissection is made using CT scan. In 1988, ultrasound was first used in the emergency department (ED) to evaluate for AAA.^17^ Bedside ED ultrasound has been shown to significantly reduce the time to diagnosis of AAA with 97.5–100% sensitivity and 94.1–100% sensitivity.^18^, ^19^ Bedside ultrasound is increasingly used to quickly diagnose aortic dissections. Gibbons *et al*.^20^ found that combining transthoracic and abdominal ultrasound resulted in the correct diagnosis of 96.4% of aortic dissections that were ultimately found on CT scan.

The use of point‐of‐care ultrasound is expanding to nearly all medical specialties.^21^, ^22^, ^23^, ^24^ This necessitates an increase in ultrasound training during residencies and medical school. Ultrasound phantoms are often utilised during educational sessions to demonstrate normal findings and pathology. A commercially available aortic aneurysm phantom exists, but there is not currently a phantom that demonstrates aortic dissection. Commercial phantoms can be expensive; therefore, many ‘homemade’ phantoms have been developed to demonstrate a variety of conditions and for use as procedural trainers.^25^ We have created phantoms that mimic aortic aneurysm and aortic dissection that are inexpensive and easy to construct.

## Materials and methods

**Figure 2:**
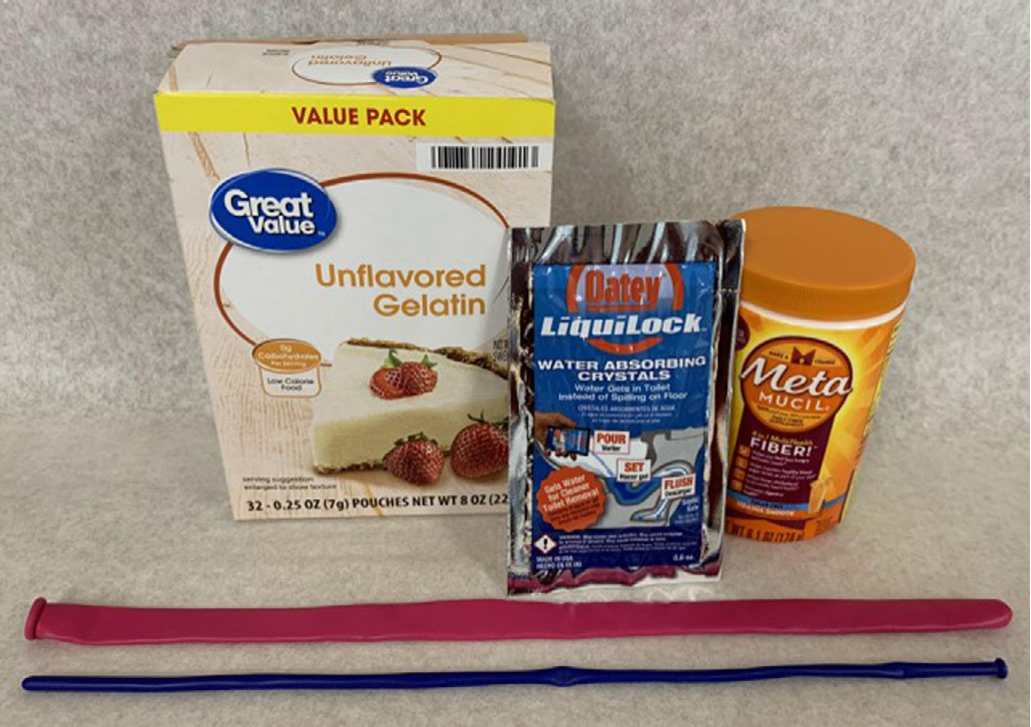
Supplies needed for making the aortic phantoms: 160 and 360 balloons, water‐absorbing gel crystals, unflavored gelatin and sugar‐free psyllium hydrophilic mucilloid fibre. [Colour figure can be viewed at wileyonlinelibrary.com]

The phantoms were made using size 160 and 360 balloons (Sempertex, Barranquilla, Colombia), water‐absorbing gel crystals (Liquilock, Oatey, Cleveland, OH), unflavored gelatin (Equate, Bentonville, AR), sugar‐free psyllium hydrophilic mucilloid fibre (sugar‐free Metamucil; Proctor & Gamble, Cincinnati, OH), water and containers of desired sizes (Figure 2). We found that using a 3‐way stopcock with a length of IV tubing and a 60‐mL syringe significantly simplified adding water to the balloons when more than 60 mL of water was used. In order to completely embed the aortic aneurysm phantom in gelatin, we recommend that the container be at least 10‐cm deep; the length and width of the phantom can be modified to fit the container. We used a small cardboard box lined with a plastic garbage bag. The aortic dissection phantom needs a minimum depth of 4 cm.

### Aortic aneurysm

To make the aneurysm phantom, the balloons were cut to the desired length (~3–4 cm for our phantom), and the cut end was tied. Next, 1/8 teaspoon (5 g) of water‐absorbing crystals was added to the 360 balloon. A small funnel or a piece of straw inserted into the mouth of the balloon can facilitate this step. Then, the 160 balloon was inserted into the 360 balloon. The 160 balloon was inflated with ~60 mL of water. Care was taken to ensure that the inflation of the 160 balloon occurred within the 360 balloon as opposed to at the end of the 160 balloon near the syringe. Care was taken to ensure that all air bubbles were removed and the 160 balloon was tied. The remaining tail was cut off, and the tied end was pushed into the 360 balloon. Next, the 360 balloon was filled with ~250 mL of water. Again, care was taken to remove all air bubbles, and the balloon was tied. The water‐absorbing crystals were distributed throughout the balloon by inverting the construct multiple times and kneading it if necessary, and the remaining uninflated end was tied close to the simulated aneurysm and the ends were trimmed. If possible, we recommend tying any uninflated 160 balloon tail into the 360 balloon knot to hold it in place.

Once the aortic aneurysm was constructed, it was submerged in water and imaged with ultrasound to ensure a satisfactory appearance. It was then embedded in a gelatin–psyllium hydrophilic mucilloid fibre mixture. The mixture was created as previously described.^26^ Briefly, 1 tablespoon (8 g) of gelatin per 100 mL was suspended in water, then heated to dissolve. Once the gelatin was dissolved, 1 tablespoon (14 g) of fibre powder per 100 mL was added and stirred until evenly distributed. The mixture was then poured into the desired container. We recommend constructing this phantom in layers. We first poured a layer of gelatin–fibre mixture at least 2‐cm thick and refrigerated until set. Then, the aneurysm construct was placed on top of it and enough gelatin–fibre mixture to cover it by at least 2 cm was poured over it and refrigerated. The phantom is ready for use once the gelatin is firm. It is not necessary to remove the phantom from the container prior to use, but if removal is desired, we recommend lining the container with plastic wrap or a plastic garbage bag to ensure easy removal.

### Aortic dissection

A 360 balloon was filled with enough water to create a vessel of the desired size (~150 mL for our phantom). Air bubbles were removed, and the end of the balloon was tied. We recommend squeezing the water toward the center of the balloon and tying both ends of the balloon near the expanded section, leaving two deflated tails. The balloon was then embedded in the gelatin–fibre mixture. For this mixture, the amount of gelatin used was doubled [2 tablespoons (16 g) per 100 mL] to make a firmer phantom. Care must be taken to ensure that the balloon is completely embedded in the mixture and not be visible from any side. The balloons tend to float in the gelatin–fibre mixture, so we lined a container with plastic wrap and glued the deflated tails to the plastic leaving enough slack to allow the balloon to float at least 1 cm off the bottom of the container, ensuring that the water‐filled balloon will not float to the top of the gelatin–fibre mixture and will also not be touching the bottom of the container. Alternatively, one could create the phantom in layers as with the aortic aneurysm phantom described above and use small pieces of bent wire to secure the vessel to the base gelatin layer before adding a second layer of gelatin–fibre mixture. The phantom was refrigerated until firm. A 22‐gauge needle was then inserted into the phantom at one end to pop the balloon. Because firmer gelatin was used, the water from the balloon remains in the space previously occupied by the balloon and mimics a vessel. The deflated balloon mimics an intimal flap.

## Results

**Figure 3:**
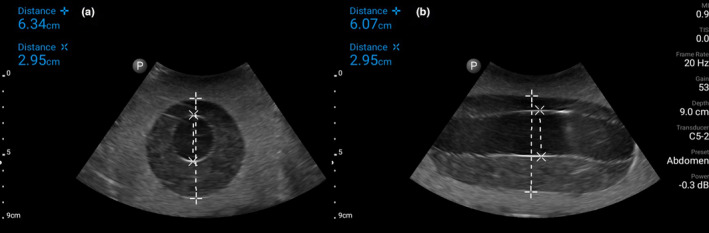
Ultrasound image using a low‐frequency curvilinear probe of the aortic aneurysm construct in gelatin mixture in transverse (a) and longitudinal (b) orientation with measurements. [Colour figure can be viewed at wileyonlinelibrary.com]

**Figure 4:**
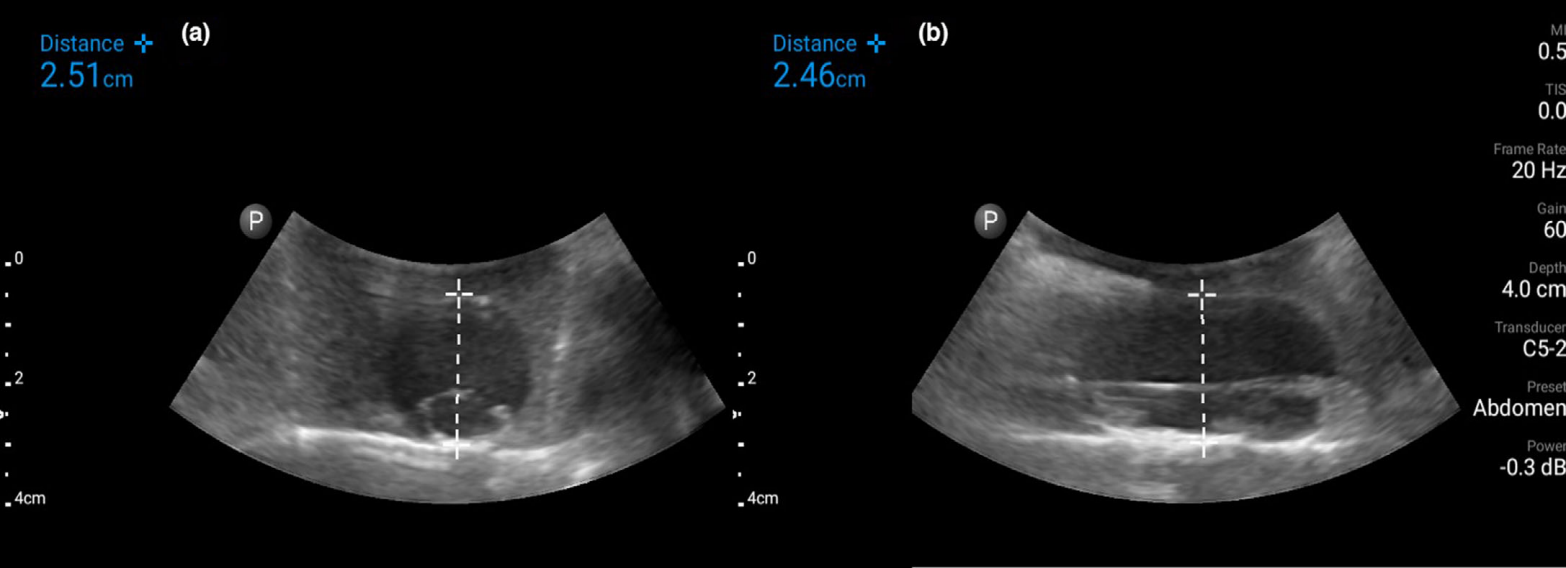
Ultrasound image using a low‐frequency curvilinear probe of the aortic dissection construct in gelatin mixture in transverse (a) and longitudinal (b) orientation with measurements. [Colour figure can be viewed at wileyonlinelibrary.com]

These models realistically mimic aortic aneurysm (Figure 3) and aortic dissection (Figure 4). The 160 balloon has a diameter of ~3 cm when inflated, which is similar to a normal aorta. The 360 balloon diameter can reach over 6 cm, which accurately simulates an aortic aneurysm. The water‐absorbing crystals in the 360 balloon simulate a mural thrombus. This construct reinforces the need to measure the aorta from outer wall to outer wall, rather than measuring just the luminal diameter. In the aortic dissection phantom, the use of a more concentrated gelatin mixture keeps the water from the deflated balloon in the cavity formed around the previously inflated balloon and the deflated balloon floats in the water and mimics an intimal flap. If the gelatin surrounding any part of the phantom is not thick enough, the water will leak out once the balloon is deflated.

These phantoms constructs take only a few minutes to make. Embedding them in layers of the gelatin–fibre mix increases the creation time depending on the depth of gelatin needed and how many layers are used. In general, each gelatin layer takes 2–3 h to solidify when refrigerated. The estimated cost for these phantoms is under $30 USD. Once purchased, the materials can be used to create multiple phantoms. The brands used in our phantom may not be available locally, but other brands are not likely to significantly alter the sonographic appearance of the phantom.

## Discussion

We have created inexpensive ultrasound phantoms that demonstrate aortic aneurysm and aortic dissection. A commercial aortic aneurysm phantom exists, but there are not currently any ‘homemade’ aortic aneurysm or aortic dissection phantoms. Because they are inexpensive and easy to create, these phantoms can be incorporated into ultrasound training sessions giving learners the ability to see aortic pathology prior to scanning a live patient.

As described, the aortic aneurysm phantom represents a fusiform aneurysm. The water‐absorbing crystals in the 360 balloon creates internal echoes mimicking intramural thrombus. The use of the inner balloon with a diameter similar to a normal aorta reinforces the concept that the sonographer must measure the aorta from outer wall to outer wall when assessing the true diameter. The diameter of the aneurysm can be varied by using different amounts of water in the 360 balloon. It may be possible to create a saccular aneurysm by manipulating the water added to the 360 balloon, but we were unsuccessful.

When making the aortic dissection phantom, we recommend using as long a length of balloon as possible. When a short phantom is made, the ends of the balloon become visible once it is popped. Use of a longer balloon results in a longer length of phantom that realistically simulates an intimal flap. When making the dissection phantom, it is of utmost importance that the gelatin mixture is very dense, the entire balloon is surrounded by gelatin and a very thin needle be used to puncture the balloon. Otherwise, the water will leak out of the phantom, and it will be unusable.

There are limitations to these phantoms. Their creation requires a source of heat and access to refrigeration. They can be messy to create. As with all gelatin‐based phantoms, they have a limited shelf‐life. With refrigeration, they can last up to 7 days before growing mould or drying out. Creating the phantom in a container with an airtight lid can extend the life span from 10 to 14 days. As with any phantom, imaging is far easier and pathology more easily recognisable on these models than on patients.

## Conclusion

These aortic phantoms are inexpensive and easy to construct. They offer learners the ability to evaluate aortic pathology in a simulation setting prior to performing an aortic ultrasound on a live patient.

### Author Contribution

The authorship listing conforms with the journal's authorship policy, and all authors are in agreement with the content of the submitted manuscript.

## Funding

No funding information is provided.

## Conflict of interest

No financial support of conflicts of interest.
